# Chromatin Remodeling, DNA Damage Repair and Aging

**DOI:** 10.2174/138920212803251373

**Published:** 2012-11

**Authors:** Baohua Liu, Raymond KH Yip, Zhongjun Zhou

**Affiliations:** 1 Shenzhen Institute of Research and Innovation, The University of Hong Kong, Shenzhen, China; 2 Department of Biochemistry, LKS Faculty of Medicine, The University of Hong Kong, Hong Kong

**Keywords:** Aging, Longevity, Epigenetics, Sirtuins, Histone modifications, DNA double strand break repair.

## Abstract

Cells are constantly exposed to a variety of environmental and endogenous conditions causing DNA damage, which is detected and repaired by conserved DNA repair pathways to maintain genomic integrity. Chromatin remodeling is critical in this process, as the organization of eukaryotic DNA into compact chromatin presents a natural barrier to all DNA-related events. Studies on human premature aging syndromes together with normal aging have suggested that accumulated damages might lead to exhaustion of resources that are required for physiological functions and thus accelerate aging. In this manuscript, combining the present understandings and latest findings, we focus mainly on discussing the role of chromatin remodeling in the repair of DNA double-strand breaks (DSBs) and regulation of aging.

## 1 CHROMATIN STRUCTURE

The fundamental units of chromatin are the nucleosome core particles consisting of 147 base pairs (bp) of DNA wrapped around a histone octamer which is comprised of a central (H3-H4)2 tetramer flanked by two H2A-H2B dimers, in 1.65 left-handed helical turns [1-3]. By histone deposition onto DNA, strings of 10nm nucleosome arrays are formed. Further packing and condensation of these nucleosome arrays through histone chaperones and chromatin remodelers initiate the hierarchy of chromatin assembly from 30 nm chromatin fiber to chromatin and culminate in chromosomes. Besides the canonical histone proteins (those that constitute nucleosomes), there exist histone variants which are isoforms of histone molecules, and they are deposited onto DNA in a sequence, and chromosomal region, specific manner to fulfill their distinct functions [4, 5]. Of note, H2A variants are the most investigated histone variants due to their pivotal roles in mediating DNA damage response and in regulating chromosome stability [6-8].

## 2 CHROMATIN REMODELING

The intricate packaging of eukaryotic DNA into chromatin represents a natural barrier to all nucleotide-based processes that require recruitment of enzymes and regulatory proteins to their sites of action. To allow critical cellular processes such as transcription, differentiation and DNA repair, the chromatin must be remodeled in a coordinated manner at specific regions for the loading of reaction factors. In eukaryotes, histone-modifying enzymes and ATP-dependent chromatin remodeling complexes are two predominant factors employed to accomplish this remodeling process.

### 2.1 Histone Modifying Enzymes

Each core histone protein contains a flexible N-terminal tail protruding outwards from the nucleosome surface. These exposed tails are hotspots for histone modifying enzymes to deposit (or remove) post-translational modifications (PTM), such as methylation, acetylation, ubiquitination, phosphorylation etc [9, 10]. Accordingly, modified histones may induce conformational changes of the nucleosomes and provide docking sites for regulatory elements [11]. Hence, through direct interaction with modified histone tails, downstream proteins can be recruited to targeted chromosomal regions for various cellular processes. One notable example of the myriad effector proteins is the chromatin remodeling complexes (which will be discussed later). Most, if not all, remodelers are armed with specialized recognition domains such that they are steered to specified venues to influence local chromatin architecture for physiological purposes [7, 12-14]. Indeed, many biological processes such as transcription and DNA replication utilize an array of modified histones to constitute a “histone code” of epigenetic marks for engaging multiple reaction factors [9].

### 2.2 ATP-Dependent Chromatin Remodelers

ATP-dependent chromatin remodeling complexes, also called chromatin remodelers, are large (>1 MDa) multi-subunit complexes that are highly conserved across the eukaryotic kingdom. They are characterized by the presence of a highly conserved ATPase subunit which belongs to the superfamily II helicase-related proteins. Based on their unique ATPase-flanking domains, chromatin remodelers can be further subdivided into four distinct families, i.e. SWI/SNF, ISWI, CHD and INO80 [14]. Through ATP hydrolysis, chromatin remodelers are capable in disrupting histone-DNA contacts, leading to a wide variety of structural changes in chromatin architecture like: (i) repositioning or sliding of nucleosomes on DNA to expose specific sites [15, 16], (ii) ejecting the entire histone octamer to expose DNA [17, 18], (iii) exchanging and incorporating histone variants for canonical core histones [19], and (iv) ejecting or exchanging dimers, such as H2A-H2B dimers to destabilize nucleosome and unwrap DNA [20]. Collectively, chromatin remodelers create a dynamic environment where chromatin structure can be modulated as desired for different DNA processing activities in response to appropriate upstream cues.

#### 2.2.1 SWI/SNF Family

SWI/SNF (switching defective/sucrose nonfermenting) remodelers contain a conserved ATPase domain and a set of associated subunits which define their properties. The catalytic regions of SWI/SNF remodelers contain a unique bromodomain near their C termini, which allows their recognition and binding to acetylated histone residues for targeting and retention at certain loci [21, 22]. Some SWI/SNF remodelers even contain multiple bromodomains for specific nucleosomal targeting [23]. Like other chromatin-remodeling complexes in the SWI/SNF superfamily, SWI/SNF remodelers are highly conserved across different species, including *Saccharomyces cerevisiae*, *Drosophila melanogaster* and *Homo sapiens*. Yeast contains two distinct SWI/SNF complexes based on their subunit compositions: SWI/SNF and RSC; and their human equivalents are BAF and PBAF [24, 25]. The primary function of SWI/SNF remodelers is reorganization and repositioning of nucleosome particles to promote binding of transcription factors [26], and in contrast to ISWI remodelers, they cause random positioning of nucleosomes in an array that were initially evenly-spaced [27]. Moreover, SWI/SNF remodelers exhibit the behavior of nucleosomal-ejection that can expose target DNA sites [28]. 

#### 2.2.2 ISWI Family

The ISWI (imitation switch) chromatin remodelers contain a distinctive combination of domains at their C termini: the SANT domain (ySWI3, yADA2, hNCoR, hTFIIIB) and SLIDE domain (SANT-like ISWI) [23]. In combination, these two domains constitute a nucleosome recognition unit that binds to histone tails and linker DNA which protrudes from the nucleosome. Similar to SWI/SNF family, ISWI family consists of various ISWI complexes according to their associated proteins: ISW1a, ISW1b and ISW2 in yeast and NURF, CHARC and ACF in human [29]. ISWI complexes such as ACF and CHARC have a pivotal role in facilitating chromatin assembly by promoting ordered spacing of nucleosomes along DNA templates after DNA replication. Their nucleosome organization activities also promote repression of specific genes [30]. 

#### 2.2.3 INO80 Family

INO80 (inositol requiring 80) remodelers contain a signature long insertion in the middle of their ATPase domain, thereby generating the characteristic “split” ATPase domain [31]. The INO80 family remodelers are evolutionarily conserved and they exist as INO80 and SWR1 in yeast; INO80, SRCAP and TIP60 in human [32]. INO80 complexes display diverse chromatin remodeling activities such as nucleosome repositioning and eviction, which might mask or expose specific DNA sequences for the binding of regulatory proteins to mediate various DNA processing events [33, 34]. Unique to SWR1 and SRCAP (members of the INO80 family), they are capable of mediating histone exchanges by removing canonical H2A and replacing them with a H2A variant, H2AZ, in the nucleosome [19, 35]. 

#### 2.2.4 CHD Family

CHD (chromodomain, helicase, DNA binding) family members are characterized by two signature motifs: N-terminal tandem chromodomain and a central SNF2-like ATPase domain [32, 36]. Depending on the constituent domains, CHD proteins are categorized into three subfamilies: family I (CHD1, CHD2), family II (CHD3, CHD4), and family III (CHD5-CHD9). Notably, human CHD3 and CHD4 (sometimes referred as Mi-2α and Mi-2*β*, respectively) are incorporated into a protein complex called NURD, which displays noticeable histone deacetylase and chromatin remodeling behaviors and has a pivotal role in regulating B lymphocyte differentiation [37-39]. 

#### 2.2.5 Remodeler Mechanisms

One of the key barriers that chromatin remodelers must overcome during chromatin remodeling is the disruption of histone-DNA contacts. This impediment is particularly augmented when all 14 histone-DNA contacts must be collapsed during nucleosome sliding and ejection [40]. For this reason, all chromatin remodelers are equipped with a highly conserved ATPase domain that provides them with sufficient mechanical forces to rupture histone-DNA contacts through ATP hydrolysis. However, given the fact that each remodeler alters nucleosome particles in a unique manner to achieve their biological activities, they are probably “tailored-equipped” with certain remodeling mechanisms. Nevertheless, remodelers are believed to share four major features during chromatin remodeling: first, they mediate unwrapping of DNA from the histone octamer surface at defined positions; second, DNA is lifted off from the internal surface of nucleosome particles, presumably by altering the histone octamer structures; third, they eject target histone molecules or entire nucleosomes for removal or exchange; and fourth, they mediate directional DNA translocations in a cis manner on the octamer surfaces [26, 40]. 

## 3 DOUBLE-STRAND BREAK REPAIR

Cells and tissues in our body are constantly exposed to a variety of environmental and endogenous assaults that lead to damages in DNA, proteins and organelles. Of these, DNA is the most invariable and fundamental material that has to be transmitted to the next generation with high fidelity. It is estimated that one eukaryotic cell typically faces up to around 10^5^ DNA lesions per day, including strand breaks, mismatches, crosslink and base modifications etc [41]. In response to different types of DNA lesions, mainly five pathways are involved in repair, e.g. homologous recombination (HR), non-homologous end-joining (NHEJ), nucleotide excision repair (NER), base excision repair (BER) and mismatch repair (MMR) [42]. 

Amongst the various classes of DNA repair pathways, perhaps the best studied with respect to DNA lesion sensing and signaling is the DSB repair. DSBs are the most lethal assaults to the genetic materials and their efficient repair is essential for genomic integrity. Unrepaired or wrongly repaired DSBs could give rise to chromosomal mutations and translocations which may induce tumor formation. DSBs may also arise in response to environmental challenges such as ultraviolet light as well as endogenous processes, such as stalled DNA-replication forks, reactive oxygen species and programmed V(D)J recombination in the immune system [43, 44]. Eukaryotic cells have evolved at least three types of repair pathways to detect and correct DSBs, including HR, NHEJ, and alternative NHEJ (alt-NHEJ) pathway. HR requires the proximal existence of an undamaged sister chromatid or chromosomal homologue as a template for repairing the breakage sites, and hence is preferentially employed during late S-G2 phase when homologous chromosomes come in close vicinity [44]. HR is a high-fidelity repair process, and it requires significant chromatin remodeling because of the involvement of extensive homologous sequences. In contrast, NHEJ involves direct re-ligation of broken ends without regard to sequence homology, and hence is not restricted to any particular cell cycle phase. However, it is often an error-prone process that can result in chromosomal aberrations. As homologous sequences are not required for NHEJ, it is the predominant repair pathway in G1 and quiescent cells.

### 3.1 Homologous Recombination Repair

During HR, broken DNA ends are first recognized by the Mre11-Rad50-Xrs2 (MRX) complex (Mre11-Rad50-Nbs1 (MRN) complex in mammals) and are processed by MRE11 to 3’ single-strand DNA (ssDNA) tails through a series of 5’→3’ strand resection activities [6, 9, 45]. The C-terminus of NBS1 interacts with ATM and recruits it to DSBs [46]. ATM belongs to the phosphatidylinositol-3-like kinase-related kinase (PIKK) family and plays an important role in the propagation of the initial DSB lesion by phosphorylating a number of downstream substrates. In undamaged cells, ATM forms inactive dimers or multimers. Upon induction of DSBs, ATM is autophosphorylated at serine 1981, leading to its dissociation into activated monomers [47]. Activated ATM rapidly phosphorylates and activates downstream repair factors to directly promote their recruitment to sites of DNA damage. Perhaps, the most important event is the ATM-dependent phosphorylation of the histone variant H2AX at the C-terminal of the protein, corresponding to Ser139 (γ-H2AX) [48]. Other substrates for activated ATM include the proteins SMC1, NBS1, CHK2, p53, BRCA1 and MDC1 [49]. Key amongst these substrates are the Chk2 kinase and p53 which act to reduce cyclin-dependent kinase (CDK) activity and arrest cells in the various stages of cell cycle to allow time of the completion of DNA repair. Following recruitment and activation of ATM, BRCA1, BRCA2 and RAD52 epistasis group proteins including XRCC2, XRCC3, RAD51B, RAD51C and RAD51D [50] are also recruited to DSBs to further transmit signals to downstream processing enzymes. The single-strand overhangs are then rapidly bound by ssDNA-binding protein replication protein A (RPA), and recruit Rad51 and Rad52 to the damaged sites [45]. Loading of Rad51 onto the ssDNA tail subsequently results in the formation of ssDNA-Rad51 nucleoprotein filament, which then searches for its homologous counterpart in the corresponding intact sister chromatid. If the specific region of duplex DNA is found, strand invasion is initiated in the presence of another set of HR facilitating proteins (Rad54, Rad55, and Rad57) followed by strand exchange and joint molecule formation [45, 51]. Once the Holliday junctions are resolved, distal broken ends are sealed through DNA synthesis by DNA polymerase, resulting in an error-free repair event and preserving genetic contents [44].

### 3.2 Non-Homologous End Joining

In NHEJ, DSB ends are recognized and bound by an end-binding heterodimer consisting of Ku70 and Ku80. Together with a DNA-PK catalytic subunit (DNA-PKcs), KU protein forms a complex known as DNA-PK [9]. Their principal activity is to hold the two ends in close proximity for direct end-to-end religation by the DNA ligase 4 (Lig4) and ligase-interacting factor 1 (Lif1) in yeast or XRCC4 in mammals. Alternatively, ends bound with KU complex could be resected by MRX (MRN in mammals) complex. Similarly, processed ends are joined directly by the action of Lig4-Lif1 (or Lig4-XRCC4 in mammals) mediated ligation, creating the repaired duplex DNA [43]. However, NHEJ is an error-prone process in comparison with the HR. 

### 3.3 Alternative NHEJ

When classical NHEJ pathway is inhibited, an alternative end-joining pathway operates in cells, called alternative NHEJ (alt-NHEJ), or microhomology-mediated end joining (MMEJ) [52]. This pathway functions even in the absence of classical NHEJ factors such as Ku, XRCC4 or DNA ligase IV. For example, the alt-NHEJ pathway is robustly activated in mice lacking X4-L4 (a complex containing XRCC4, DNA ligase IV and XLF). Alt-NHEJ is mediated by the annealing of ssDNA microhomology regions followed by LIG3-dependent DNA end ligation [53, 54]. Microhomologies are short stretches of complementary “microhomology” sequences (1–10 base pairs) that often appear at repair junctions [52]. As back-up version, alt-NHEJ pathway is more error-prone than classical NHEJ and frequently results in small deletions/insertions around the region of DSBs or could even result in deleterious translocations [55].

### 3.4 V(D)J and Class Switch Recombination

The DNA damage response pathway is not just activated in response to genotoxic stress, but is also essential for normal physiological processes, such as B cell lymphogenesis. B cells are one of the main components of the adaptive immune system and are responsible for the generation of B cell receptors (BCRs, also known as immunoglobulins), which recognize a large repertoire of antigens. The mammalian BCR locus contains a large number of variable (V), diversity (D), joining (J) and constant (C) segments. V, D and J fragments are separated by recombination signal sequences (RSSs). Rag-1 and Rag-2 recognize RSSs and generate DSBs between two adjacent coding segments. Different V, D and J segments undergo rearrangement by processes termed as V(D)J recombination [56]. The proteins involved in NHEJ, including DNA-PK, Artemis, and XLF (Xrcc4-like factor, also known as Cernunnos)-Xrcc4-DNA ligase IV complex, are important for V(D)J recombination. DNA-PKcs deficiency is the main cause of murine severe combined immunodeficiency (SCID), where both B and T cells are depleted [57]. Any defects in Ku protein also impair V(D)J recombination [58, 59]. Artemis is thought to open the closed hairpin at the coding ends generated by Rag-1/2. Null mutants of Artemis also give rise to the severe combined immunodeficiency (SCID) phenotype [60]. Xrcc4 together with XLF and DNA ligase IV ligates the broken ends together. Cells deficient for either Xrcc4, XLF or DNA ligase IV are sensitive to γ-irradiation and are compromised in V(D)J recombination [61, 62].

In response to antigen or humoral stimulation, class switch recombination (CSR) further diversifies antibodies by switching their isotypes [63]. CSR occurs between two switch (S) regions located upstream of C_H_ (constant regions of immunoglobulin heavy chain). Similar to V(D)J recombination, CSR also involves DSB generation and NHEJ. Upon humoral stimulation, activation-induced cytidine deaminase (AID) deaminates deoxycytidine (dC) resulting in deoxyuracil (dU) bases on both strands of two transcriptionally active S regions [64]. The dU is excised by the uracil DNA glycosylases (UNG) and the resultant abasic site is further cut by apurinic/apyrimidinic endonuclease 1/2 (APE-1/2), generating single strand breaks (SSBs). Either two adjacent SSBs on opposite strands spontaneously lead to one DSB, or the MMR machinery is triggered to convert SSB to DSB [65]. Deficiency of AID, UNG, APE or any of the MMR components, including Msh2, Msh6, Mlh1, Pms2 and Exo1, leads to loss or reduction of CSR in B cells [63]. After DSB formation, the NHEJ pathway is activated. The Ku70-Ku80 heterodimers bind to the DNA ends and recruit necessary proteins to process the DNA ends to facilitate the ligation mediated by Xrcc4-DNA ligase IV complex [66]. CSR in *Ku70^─/─^* and *Ku80^─/─^* B cells is nearly ablated [67, 68]. Either Xrcc4 or DNA ligase IV deficiency causes significant reduction in CSR [69, 70]. While compatible ends are joined rapidly by canonical NHEJ components, complex lesions need substantial processing and are re-ligated slowly. In the later case, ATM, 53BP1 and MRM complex cooperate with canonical NHEJ components to mediate end-joining recombination. Disruption of ATM, 53BP1 or MRN complex in mice leads to defects in either V(D)J recombination or CSR or both [71-74]. Recent studies in mouse models deficient in NHEJ core components revealed a robust alt-NHEJ pathway that utilizes microhomology to mediate the end joining in CSR [69, 70]. Alt-NHEJ leads to Ig locus deletion and translocation. However, the molecular mechanisms underlying alt-NHEJ are not well elucidated so far.

### 3.5 Meiotic Recombination

Another example of a physiological DNA damage response is meiosis. Meiosis is a form of cell division occurring in sexually reproducing organisms by which maternal and paternal chromosomes are distributed between cells to generate genetic diversity. Prior to meiosis, each chromosome duplicates and creates two sister chromatids, which stay connected at the centromere. During meiosis, the homologous chromosomes align in parallel and chromosomal crossovers are induced by recombination. DSBs are generated by meiosis-specific topoisomerase-like enzyme Spo11 [75], together with Mei1. Mice lacking Spo11 or Mei1 fail in the generation of DSBs, leading to absence of Rad51 foci, faulty synapsis, meiotic failure and eventually infertility [76-79]. Many components of the HR pathway are of particular importance for proper strand exchange and meiosis. MRN complex is recruited to the DSBs to remove Spo11 and degrade the 5’ of DNA, generating long 3’ ssDNA overhangs. ATM is activated by MRN and further amplifies the signaling via phosphorylation of downstream transducers and effectors, such as H2AX and Chk2 [80]. Finally, Rad51 and meiosis-specific Dmc1 recognize and bind to the resected 3’ ssDNA overhangs and form nucleoprotein filaments, which mediate the search for homologous template and subsequent strand exchange. As they are all essential for development, disruption of either Rad51 or any component of MRN complex causes embryonic lethality [81-83]. Loss of ATM in mice causes general defects in DSB repair and mislocalization of Rad51 and Dmc1 in spermatocytes [84, 85]. During meiosis, *Dmc1^─/─^*germ cells get arrested at the early zygotene stage due to the failure of homologous chromosome synapsis [86]. 

## 4 CHROMATIN REMODELING IN DSB REPAIR

It is becoming increasingly clear that in response to DSBs, compact chromatin, especially heterochromatin, needs to be opened up for the loading and retention of necessary repair proteins to the DNA lesions, which is called chromatin remodeling [87]. As described above, chromatin remodeling is regulated by multiple and integrated mechanisms including covalent histone modifications, non-histone chromatin mediators, ATP-dependent chromatin remodelers etc [10, 88]. 

### 4.1 Covalent Histone Modifications

One of the earliest histone modifications in response to DSBs is the phosphorylation of mammalian H2A-variant H2AX at the carboxy-terminal serine 139 tail (γ-H2AX) by ATM [43]. Within seconds, γ-H2AX spreads from the initial break to a large region (tens of kilobases in yeast and up to millions of base pairs in mammalian cells) and creates a specialized chromatin compartment capable of recruiting and retaining DNA repair factors [43, 51, 89]. Amongst the various proteins recruited by γ-H2AX, MDC1 is particularly important. Accumulation of several other repair factors such as NBS1, 53BP1 and the phosphorylation of ATM are reduced when the MDC1-γ-H2AX interaction is abrogated. Upon the generation of γ-H2AX, MDC1 together with MRN are recruited via the BRCT domain of MDC1. The binding of ATM to MDC1 and MRN further promotes the phosphorylation of H2AX, resulting in the amplification of the DNA damage response [90].

Recently an important breakthrough in this area of research was made upon the identification of RNF8, the first of the three E3 ubiquitin ligases that catalyze regulatory ubiquitination at DNA lesions [91]. RNF8 recognizes the ATM phosphorylated site of MDC1 and thus is recruited to the DNA lesions. Meanwhile, RNF8 interacts with a second E3 ligase HERC2, thus recruiting HERC2 to DSB sites. RNF8 together with HERC2 facilitates the assembly of the E2 ubiquitin conjugating enzyme Ubc13 to initiate K63-linked ubiquitin chains on H2A and its variants. The third E3, RING domain ubiquitin E3 ligase RNF168, recognizes and binds to K63-linked ubiquitin chains on H2A and H2AX through its two MIUs (Motif Interacting with Ubiquitin). This amplifies the local concentration of K63-linked ubiquitin resulting in the recruitment and retention of 53BP1 and BRCA1 at the sites of lesions. Interestingly RNF168 was first identified as the gene mutated in RIDDLE syndrome, a novel human immunodeficiency disorder associated with defects in DSB repair and Brca1/53BP1 recruitment defects [92].

Apart from phosphorylation and ubiquitination, histone acetylation and methylation also mediate chromatin remodeling and recruitment of repair proteins. For instance, in budding yeast, dynamic histone acetylation surrounding DSBs are required for HR [93]; in mammalian cells, histone acetyltransferase (HAT) Tip60 acetylates H2AX at K5 and thus promotes ubiquitination of H2AX at K119 and enhances chromatin remodelling [94]. In a similar manner, H4 acetylation mediated by Tip60 co-factor Trrap is required for the recruitment of DNA repair factors to DNA lesions [95]. Likewise, Mof, another MYST family HAT, mediates H4K16 acetylation, which is incorporated into nucleosomal arrays, thus impeding the formation of cross-fibre interactions and converting chromatin into a relaxed status to promote repair protein recruitment [96, 97]. Owing to global compaction of the genome, the chromatin structure of Mof-null MEFs was altered in a way such that the recruitment of repair proteins were defective and the cells became refractory to DNA damage [97, 98]. Another example is HMGN1-dependent H3K14 acetylation, which regulates ATM activation and higher order chromatin structure during DNA repair [99]. Recently, the essential roles for chromatin remodeling mediated by Tip60 [100, 101] and casein kinase 2 (CK2) [102], were further highlighted in the initial activation of DNA damage checkpoint response and repair. Upon γ-irradiation, HP1*β* is rapidly phosphorylated by CK2 and released from H3K9me3 (a pericentric heterochromatin marker) [102]. At the same time, MRN complex recruits Tip60/ATM complex to DSBs, where Tip60 binds to the exposed H3K9me3 to acetylate and subsequently activate ATM [103]. In addition to H2AX, ATM phosphorylates KAP-1 at serine 824 (p-KAP-1) [104]. KAP-1 belongs to tripartite motif family and interacts with Kruppel-associated box domain-containing transcription factors to mediate heterochromatin formation and gene silencing [105]. Upon DSBs, phosphorylation of KAP-1 spreads rapidly throughout the whole genome, triggers global chromatin remodeling and facilitates DNA repair [106]. Knocking down KAP-1, Suv39h1/2 (methyltransferases responsible for H3K9me3), HDAC1/2 or HP1α/*β*/γ (heterochromatin protein 1) rescues the defects in chromatin remodeling and DNA repair caused by the deficiency of ATM signaling [104]. Likewise, PWWP domain-containing protein EXPAND1 was shown to accumulate in DNA lesions in a H2AX, MDC1, RNF8 and 53BP1-dependent manner and increase chromatin accessibility [107]. In addition, a recent study found that the loading of 53BP1 to DNA lesions is enabled by a local increase in H4K20 dimethylation surrounding the DSBs that is catalyzed by the histone methyltransferase MMSET [108].

Another layer of evidence for DNA damage-induced chromatin remodeling comes from V(D)J and meiotic recombination. Enhancer of Zeste 2 (Ezh2) is the methyltransferase that trimethylates H3K27 (H3K27me3). Conditional deletion of Ezh2 in B lymphocytes leads to reduced H3K27me3 level and defective V(D)J recombination at the most distal V segments [109]. Similarly, di- and trimethylation of histone H3K4 are associated with active segments in V(D)J recombination [110]. Rag-2 binds to H3K4me3 via the PHD motif and mutations that abolish this interaction impair V(D)J recombination. In contrast, H3K9me2, a silent chromatin mark, inhibits distal V(D)J recombination [111], and Pax5, a transcriptional factor required for early B cell commitment, regulates the removal of H3K9me2 and promotes V(D)J recombination [112]. The generation of DSB on meiotic chromosomes is not entirely random but occurs preferentially on specific chromosomal locations, known as hot spots. Recombination regulator 1 (RCR1) and double strand break control 1 (DSBC1) regulate the activities of recombination hot spots [113, 114]. Although the molecular mechanisms underlying the selection of hot spots for DSB induction are still under investigation, it has been found that high-order chromatin structure could be an important factor [115]. Of particular interest is the methylation of histone H3K4me3 by the methyltransferase Prdm9 (also known as Meisetz), which is enriched at meiotic recombination hot spots [115-117]. Prdm9 null mice are sterile owing to defective chromosome paring and impaired sex body formation [118, 119].

### 4.2 ATP-Dependent Chromatin Remodeling in DSB Repair

#### 4.2.1 INO80

The INO80 complex has been recently implicated in DSB repair by several lines of evidence. For instance, INO80 is recruited to DSBs through its Nhp10 and Arp4 subunits, and *ino80△*, *arp8△* and *arp5△* mutants are hypersensitive to DSB-inducing agents [9, 13, 120]. Indeed, INO80 has been shown to be specifically associated with HRR by several studies. For example, mutation of Arp8, a subunit of yeast INO80 complex, reduces HR frequencies by approximately four fold compared with wild-type and also decreases formation of ssDNA by strand resection [45]. In coherence, a mutagenesis study indicates that yeast INO80 is involved in executing cell-cycle checkpoint adaptation in the early steps of HR. Meanwhile, several reports have indicated that the primary function of *S. cerevisiae* INO80 remodelers is the eviction of nucleosomes surrounding the DSBs at both homologous donor and recipient locus. This remodeling activity may facilitate strand invasion by exposing the site of DSB for assembly of HR repair and checkpoint factors such as Rad51 and Rad52 [34]. INO80-mediated nucleosome eviction also removes γ-H2AX, suggesting their role in mediating recovery from DNA damage checkpoint.

Similar to the INO80 complex, yeast SWR1 complex, another remodeler complex in the INO80 family, is also speculated to participate in DSB repair. This hypothesis is based on co-localization of this complex with γ-H2AX, albeit at lower amount than INO80, and the increased sensitivity to DNA-damage inducing agents in *swr1*Δ mutant cells [7, 19]. In contrast, the SWR1 complex displays no nucleosome eviction behavior and seemingly acts predominantly in NHEJ. The distinct contribution of SWR1 to NHEJ is also reflected by its ability to recruit Mec1 and Ku80 to DSBs [120]. 

In addition to INO80 and SWR1 complexes, a mammalian equivalent complex known as TIP60 has both acetylation and ATPase activities. Recently, a study on *Drosophila melanogaster* TIP60 demonstrated that the complex is responsible for acetylating the phosphorylated H2av (the counterpart of γ-H2AX) and subsequent exchange for an unmodified H2Av (the counterpart of H2AZ) during DNA repair. This histone exchange activity may further alter the local chromatin structure to enhance subsequent processing of DNA repair. Furthermore, growing evidence indicates that the histone acetylase activity of TIP60 and NuA4 complex, which share the actin-related protein 4 (Arp4) subunit, is required for H4 hyperacetylation at the break site [121]. This site-specific histone modification process was shown to facilitate the recruitment of certain repair factors such as Brca1 and Rad51 to DSBs lesion by generating a more relaxed chromatin structure, presumably through unpacking of higher-order nucleosomal arrangements [122]. In addition, TIP60 complex also contributes to the rapid acetylation and activation of ATM during the early phase of DNA repair. 

#### 4.2.2 SWI/SNF

The SWI/SNF complex is directly connected to DSB repair because of its recruitment to the DSBs through interaction with γ-H2AX and more recently with acetylated H3, and the fact that yeast SWI/SNF mutants displayed increased sensitivity to DSB-inducing agents [123, 124]. In particular, SWI/SNF is predominantly associated with HR because the absence of SWI/SNF complex impairs the strand invasion, and the complex associates directly with the recipient and donor loci at DSBs [125]. In contrast to mutations in INO80, Rad52 and Rad51 strand invasion proteins are recruited normally to the recipient locus in swi/snf mutants but not to the donor locus [44]. Given that molecular analysis predicts INO80 and SWI/SNF are simultaneously assembled at DSBs, current understanding suggests that INO80 is required for disrupting chromatin at the recipient locus while SWI/SNF acts on the corresponding donor locus at or prior to stand invasion [44, 45]. The nucleosomal sliding behavior of SWI/SNF *in vitro* also suggests direct intrusions around DSBs to promote assembly of HR repair factors [40, 126]. It has been recently reported that BRIT1 (BRIT-repeat inhibitor of hTERT expression) recruits SWI-SNF complex to DSBs via ATM/ATR-dependent phosphorylation of BAF170, thus regulating global chromatin remodeling [127].

#### 4.2.3 RSC

RSC remodeler was first identified in yeast as a complex closely related to SWI/SNF, and like SWI/SNF, it also contributes to both NHEJ and HR [24]. In response to DSBs, the catalytic subunits of RSC, Rsc8 and Sth1, are rapidly recruited to the DNA lesions with approximately identical kinetics as Mre11 of the yeast MRX complex, and facilitate local chromatin reconfiguration that in turn enhances accessibility of repair factors to DSBs [125, 128]. Indeed, an *in vivo* study demonstrates that RSC is loaded to the DSB and specifically interacts with Ku80, suggesting its role in mediating NHEJ repair [121]. All these findings point to the fact that the recruitment of RSC may not require H2A phosphorylation or H4 acetylation, and thus provide an alternative activation mechanism for eliciting DSB repair. 

Similar to SWI/SNF, RSC acts in HR where both RSC and SWI/SNF are recruited to the DSB, albeit with different kinetics. Thus, it is likely that SWI/SNF acts coordinately with RSC in orchestrating HR where the former is required after or before strand invasion, and RSC contributes to both the early and postsynaptic step of HR [44]. Given their intrinsic nucleosome mobilizing nature, they may facilitate the removal of nucleosomes on the donor sequence during HR to expose DNA sequence for homology-searching machineries. Interestingly, experiments in G2-phase cells demonstrated a ~50kb domain around the DSB, which was enriched with cohesion and phospho-H2A, suggesting a link between DSB repair and the cohesion accumulation [129]. Recently, growing evidence indicates that RSC is responsible for loading cohesin at DSBs to hold sister chromatids in close proximity for strand invasion and Holliday junction formation during HR [130]. Further studies revealed that the cohesion loading process requires physical interaction between RSC subunits and Mre11 for site-specific actions [128]. Thus, by loading cohesin to a specific chromosome arm during DSB repair, RSC facilitates the association of identical sister chromatids during HR and promotes direct re-ligation of proximal broken ends during NHEJ.

## 5 CHROMATIN REMODELING AND DNA REPAIR DEFECTS IN AGING

Aging involves a gradual deterioration of several physiological functions, resulting in the reduced capacity to repair injured organs, increased propensity to infections, cancer predisposition and decreased fecundity [131, 132]. Many hypotheses have been proposed to understand the underlying mechanisms of the aging process, and these include the disease theory, free radical theory and DNA damage accumulation theory. Here we only discuss evidences that support the relationship between DNA damage accumulation and aging, a concept proposed more than half century ago by Leo Szilard [133]. 

### 5.1 DNA Repair and Aging

Living cells are constantly exposed to various endogenous or exogenous conditions leading to DNA lesions that trigger DNA damage checkpoint response and DNA repair signaling. While most of the DNA damages are successfully repaired, some of them may remain unrepaired and accumulate in the cells, and some may lead to detrimental gene mutations and thus cell transformation if they are aberrantly repaired. The DNA damage accumulation theory of aging posits that accumulation of unrepaired/unrepairable DNA damage within cells leads to a sustained DNA damage checkpoint response and induces a state called cellular senescence, wherein cells permanently exit from the cell cycle, eventually leading to organismal ageing [134, 135]. Consistent with this idea, it has been documented that DNA damage in the form of DSB-specific foci containing γ-H2AX accumulate in senescent human cells, germ and somatic cells of aged mice, and in dermal fibroblasts from aged primates [136]. Mouse models harboring deficiency in DNA repair proteins, such as ATM, Ku70, Ku80, DNA ligase IV or Ercc1 also show premature aging phenotypes, providing evidence of a direct correlation between impaired DDR and premature aging [137, 138]. 

The relationship between DNA damage accumulation and aging has gained maximum credibility through studies conducted on various human progeria syndromes, which are genetic disorders where patients precociously develop features resembling natural aging. Most of the reported progeria syndromes, including Werner syndrome (WS), Bloom’s syndrome (BS), Rothmund-Thomson syndrome (RTS), Cockayne syndrome type A and type B (CSA and CSB), Xeroderma pigmentosum (XP), Trichothiodystrophy (TTD) and Hutchinson-Gilford progeria syndrome (HGPS) are caused by mutations of genes that are directly or indirectly involved in DNA repair. Of these, WS, BS and RTS are associated with defects in RecQ helicases, i.e. RECQL2 (WRN), RECQL3 (BLM) and RECQL4 respectively, whereas CS, XP and TTD shared similar defects in NER pathway. RecQ helicases are a group of highly conserved proteins from bacteria to humans. The roles of RecQ helicases in DNA metabolism, including DNA replication, transcription, repair and recombination, have been extensively investigated and are demonstrated to be the underlying pathological basis of WS, BS and RTS [139-142]. Most recently, delayed DNA damage checkpoint response and defective DNA repair were found to contribute to the progeria phenotypes in HGPS as well [143].

#### 5.1.1 Werner Syndrome

WS is an autosomal recessive genetic disorder of “progeria in adulthood”, affecting about 10 in one million [144]. Patients suffering from WS are usually born healthy with obvious growth retardation from the second decade and other ageing-related features, including short stature, premature cataract, beaked nose, skin atrophy and alopecia, loss of adipose tissues, type II diabetes, osteoporosis, arteriosclerosis, hypogonadism and predisposition to cancer. WS patients typically die of early onset cardiovascular diseases or neoplasia in the fourth decade of life and have an average life expectancy of 47 years. Since WS closely resembles physiological aging, WS cells have been the subject of intense investigation to understand the biology and molecular mechanism of normal aging. Skin fibroblasts cultured from affected individuals develop accelerated senescence with increased chromosome aberrations [145, 146]. By positional cloning, WRN was firstly linked to WS [147]. Before the identification of *LMNA* mutations (see below) in atypical WS, WRN was the only protein implicated in WS. WRN belongs to the family of RecQ helicases and is the only member with a specific exonuclease domain within the N-terminus [148, 149]. Physiological and functional interactions between WRN and other proteins suggest that it has crucial roles in DNA replication and repair. WRN interacts with proteins required for DNA replication, such as RPA, PCNA, FEN1 and DNA polymerase (Polδ) [139, 150-152]. Studies from Lebel and colleagues indicated that WRN was involved in restoration of stalled replication forks. WRN interacts with NHEJ thus regulating DSB repair [141, 142, 153]. WRN also associates with three of the six members of telomere complex, including telomeric repeat binding factor 1/2 (TRF1/2) and POT1, to maintain telomere integrity [144, 154]. Shortened or uncapped telomeres can be recognized as DSBs and thus trigger DDR, leading to end-to-end fusions [155]. Telomere shortening is one of the most important causes of replicative senescence [156]. In a recent study, Kusumoto-Matsuo and colleagues showed that DNA-PKcs interacts with WRN thus stimulating its helicase activity while preventing the exonuclease digestion of telomeric D-loop [157]. Recently, lamin A/C mutations (A57P, R133L, L140R, and E578V) were also reported in autosomal dominant atypical WS where patients presented with more severe phenotypes compared to those associated with WRN [158-160]. 

#### 5.1.2 Bloom’s Syndrome

Bloom’s syndrome is characterized by dwarfism, sun-induced erythaema, type II diabetes, narrow face and prominent ears, infertility, and benign and malignant tumors. Deaths usually result from neoplasia before the age of 30. RecQ helicase BLM is associated with BS and has been shown to regulate HR. Upon DNA damage, BLM forms discrete nucleoplasmic foci that co-localize with RAD51 as well as BRCA1-associated genome surveillance complex (BASC) containing BRCA1, MLH1, MRN complex and ATM in mammalian cells [161]. BLM is also involved in the correct localization and activation of topoisomerase IIIα[162]. Deletion of Blm in mice results in early embryonic death by E13.5. Blm mutant embryos show growth retardation and *Blm^─/─^* ES cells have an elevated frequency of HR between sister chromatids [163, 164]. Goss *et al.* showed that haploinsufficiency of Blm caused early development of lymphoma [165].

#### 5.1.3 Hutchinson-Gilford Progeria Syndrome

HGPS is an extremely rare severe genetic disorder of early onset premature aging, also referred to as “progeria in childhood”. So far, only about 100 patients have been reported, mainly in the western world. Patients with HGPS can only survive for 12-16 years with a mean age of 13.4 years and are clinically characterized with early growth retardation, short stature, lipodystrophy, alopecia, stiff joints, osteolysis, dilated cardiomyopathy and atherosclerosis [166, 167]. A recurrent *de novo* dominant point mutation (1824 C→T) of the *LMNA* gene was identified to be responsible for about 76% of reported cases of HGPS. Lamin A is firstly synthesized as prelamin A with a C-terminal CAAX motif. ZMPSTE24, a metalloprotease, is required for Lamin A maturation [168]. The mutation that causes HGPS activates a cryptic splicing donor signal in exon 11, leading to 150 nucleotides deletion in mutant transcript and a 50-residue truncation in prelamin A protein. This truncated form of prelamin A, referred to as progerin, lacks the second proteolytic cleavage site of ZMPSTE24 but retains the CAAX motif [169, 170]. It was further demonstrated that around 80% transcripts from the mutant allele encodes progerin [170]. Human cells engineered to express progerin have proliferation defects and premature senescence [171, 172]. Mice deficient for Zmpste24 also manifest many progeroid features found in HGPS patients [173]. Reduction of the prelamin A level in *Zmpste24^─/─^* mice by *Lmna *heterozygosity ameliorates progeroid phenotypes and extends lifespan [143, 174, 175]. 

One of the hallmarks of HGPS cells is a misshaped nucleus, which leads to disorganized heterochromatin [143, 173, 176] and mislocalized nuclear proteins [177]. It has been shown that treatment with farnesyl transferase inhibitor (FTI) rescues the abnormal nuclear shape and thus alleviates progeroid features in both HGPS cells [178-180] and mouse models [179, 181, 182]. The rationale behind FTI treatment is largely based on the notion that the farnesylated carboxyl tail tethers prelamin A or progerin onto the nuclear lamina and thus jeopardizes the nuclear shape, and FTI treatment reduces farnesylation of prelamin A or progerin. However, this mechanistic explanation has been recently challenged by data that nonfarnesylated progerin also causes misshapen nucleus and progeroid phenotypes in mice [183]. In addition, direct expression of mature lamin A (18 a.a.-deleted), bypassing the complicated four steps of prelamin A processing, also deteriorates the nuclear shape but does not accelerate aging process in mice [184]. An alternative mechanism is suggested by independent studies revealing that accumulation of progerin and unprocessed prelamin A leads to either delayed or reduced recruitment of DNA repair proteins to sites of DSBs in HGPS cells and mice lacking Zmpste24 [143, 185-188]. 

### 5.2 Chromatin Remodeling in Aging

It has been proposed for a long time that chromatin undergoes repressive remodeling during senescence [189]. For instance, H4K20me3 increases with age in rat kidney and liver [190]. H3K9me3 and heterochromatin protein 1 (HP1) increases and redistributes in aged *Drosophila melanogaster*, leading to global alterations in aging-related gene expression [191]. Consistently, oncogene-induced senescence (OIS) induces the formation of facultative heterochromatin domains, known as senescence-associated heterochromatin foci (SAHF) [192-194]. SAHF are enriched with H3K9me3 and HP1, while excluding active histone modifications such as K3K9ac, and usually cause a stable repression of E2F target genes [194, 195]. H3K9me3 marks constitutive heterochromatin and is mainly mediated by Suv39h1/2 methyltransferase [196]. The critical roles for Suv39h1/2 and H3K9me3-mediated chromatin remodeling in aging are highlighted by the findings that loss of Suv39h1 ablates OIS in tumor mouse model [197], while Suv39h1 overexpression leads to growth retardation [198]. 

Similarly, H3K27me3, mediated by Ezh2 methyltransferase and JMJD3 and UTX-1 demethylases, normally marks facultative heterochromatin and suppresses the Ink4a-Arf locus in young dividing cells; however, in RAS-induced senescence, Ezh2 goes down whereas JMJD3 goes up, leading to upregulation of p16INK4A and p14ARF in human diploid cells [199, 200]. Knocking down *utx-1* in *C. elegans* modulates insulin/IGF-1 signaling (IIS) pathway and extends lifespan [201]. JMJD3 and UTX-1 also regulate demethylase- independent chromatin remodeling via SWI/SNF remodeling complex [202], highlighting critical roles for chromatin remodeling in the aging process. Another member of polycomb complex, Bmi1 works closely with Ezh2 to inhibit p16Ink4a expression, thus preventing adult stem cell exhaustion [203]; meanwhile it regulates H2A ubiquitination in response to DNA damage thus maintaining genome integrity [204]. In contrast to the seemingly contradictory effects of H3K9me3 in oncogene-induced senescence, normal aging and HGPS, the level of H3K27me3 is down-regulated in fibroblasts derived from patients with HGPS [205].

The most direct evidences that chromatin remodeling defects might trigger aging process come from the studies of Sirtuins, class III histone deacetylases (HDACs), containing NAD+-dependent protein deacetylase and ADP-ribosyl transferase activities [206, 207]. Of seven mammalian Sirtuins, SIRT1 is the closest homolog of *S. cerevesiae* Sir2 (silent information regulator 2), identified about three decades ago [208]. Sir2 mediates the formation and maintenance of heterochromatin in regions adjacent to telomeres, at the silent mating type information (HM) loci, and at ribosomal DNA (rDNA) to modulate longevity in budding yeast [209]. Loss of Sirt1 in mice causes defective gametogenesis, heart and retinal abnormalities, genomic instability, small body size and reduced survival [210-212], while transgenic mice with additional copies of Sirt1 show phenotypes consistent with improved healthspan [213-216]. Resveratrol, a compound identified in a screen for Sirt1 activators [217], has been reported to enhance healthspan in a range of age-related disease assays. While evidence that resveratrol directly activates Sirt1 has been called into question [218-221], many of its *in vivo* benefits are dependent on Sirt1 [222]. In addition to histones, SIRT1 deacetylates a variety of proteins, thus regulating various cell functions, e.g. genomic integrity, the inflammatory response, glycogenesis, adipogenesis, mitochondrial biogenesis and stress resistance etc [223]. Recently, it is shown that both SIRT1 protein level and deacetylase activity declines when cells are getting old [224, 225], whereas ectopic SIRT1 eliminates SAHF and prolongs replicative life-span in human diploid cells [226]. 

SIRT6 is another mammalian Sirtuin that predominantly localizes in the nucleus. Loss of Sirt6 in mice causes defective BER and accelerates aging [227]. In contrast to the diverse substrates of SIRT1, acetylated H3K9 and H3K56 are the only reported deacetylation targets of SIRT6 so far [228]. SIRT6 associates with telomeres and deacetylates H3K9ac, thus stabilizing WRN, which is mutated in WS [229]. SIRT6 also interacts with NF-κB RELA subunit and deacetylates H3K9ac in its target promoter region, thus inhibiting NF-κB signaling [230]. Recently, it was found that SIRT6 deacetylates H3K56ac [231, 232], which is important for maintaining the genome integrity [233, 234], and overexpression Sirt6 in male mice inhibits IIS pathway thus increasing life-span [235].

The critical roles for histone acetylation in aging are further emphasized by multiple acetyltransferase complexes and HDACs other than Sirtuins in multiple model systems. For example, in budding yeast, H3K56 acetylation declines with aging [236]; in aged *C.elegans*, the acetylation level of H4K5 decreases, whereas sodium butyrate (NaB) and trichostatin A (TSA) (class I and II HDAC inhibitors [237]) extend lifespan [238]; In mouse models, dysregulation of H4K12 acetylation is associated with cognitive decline along with the aging process [239].

The roles for chromatin remodeling in aging are further emphasized in progeria and other aging-related diseases. Decreased H3K9me3 but increased H4K20me3 was found in HGPS cells [176, 240]. Recently, two histone binding proteins, RBBP4 and RBBP7, were found reduced, and that is responsible for the DNA damage accumulation in HGPS cells [241]. In another study, mis-localized acetyltransferase MOF and consequent reduced acetylation of H4K16 were observed in Zmpste24 null mice [242]. Pre-incubation with NaB or TSA up-regulates global histone acetylation and feeding progeria mice with NaB rescues delayed recruitment of 53BP1, decreases senescence, and extends lifespan. It has been shown that TSA treatment activates ATM upon DNA damage [47]. Thus, in addition to local H4K16 acetylation surrounding DNA lesions, NaB/TSA treatment might enhance ATM activity thus rescuing defective global chromatin remodeling and defective DNA repair in progeria mice. HDAC inhibitors were also shown to improve DNA repair in an OIS model by causing chromatin relaxation [243]. In other studies, HDAC inhibitors have been shown to increase learning ability, delay age-dependent neurodegeneration, delay Alzheimer’s disease progression in mouse models, accelerate age-associated osteogenesis and increase lifespan of worms in a dietary restriction model [238, 244-247].

## 6 CONCLUSIONS

It is now becoming clear that chromatin conformation changes mediated by covalent histone modifications and ATP-dependent chromatin remodelers are essential for the efficient repair of DSBs, which is highly conserved across species. The chromatin remodeling and DSB repair pathways are critical for physiological V(D)J recombination, CSR, meiosis etc. Defects in these processes are associated with pathological changes during aging, as evidenced by the growing number of human premature aging syndromes and premature aging mouse models that are associated with defective DNA repair pathways. Future challenges lie in further delineating the exact mechanisms of DSB repair in the context of chromatin, how chromatin remodeling modulates human aging, and developing therapeutics for the improvement of human health and even extension of life span.
